# The extracellular matrix as an adhesion checkpoint for mammary epithelial function

**DOI:** 10.1016/j.biocel.2006.11.004

**Published:** 2007

**Authors:** Elad Katz, Charles H. Streuli

**Affiliations:** Wellcome Trust Centre for Cell-Matrix Research, Faculty of Life Sciences, University of Manchester, Manchester M13 9PT, UK

**Keywords:** Mammary, Breast, Adhesion, Integrin, Apoptosis, Anoikis, Proliferation, Differentiation, Prolactin, Rac

## Abstract

The development of the mammary gland is spatially regulated by the interaction of the mammary epithelium with the extracellular matrix (ECM). Cells receive cues from the ECM through a family of adhesion receptors called integrins, consisting of α- and β-chain dimers. Integrins assist cells in sensing their appropriate developmental context in response to both hormones and growth factors. Here we argue that cell adhesion to the ECM plays a key role in specific developmental checkpoints, particularly in alveolar survival, morphogenesis and function. Specific ablation of αβ1-integrins in the luminal epithelium of the mammary gland shows that this sub-type of receptors is required for proliferation, accurate morphological organisation, as well as milk secretion. Downstream, small Rho GTPases mediate cellular polarisation and differentiation. Current challenges in studying the integration of signals in checkpoints of mammary gland development are discussed.

## 1 The temporal–spatial nature of mammary gland development

The hormonal control of mammary gland development has been studied over several decades (Ceriani, 1974; Vonderhaar, 1988; Neville, McFadden, & Forsyth, 2002; Hennighausen & Robinson, 2005). The spatial aspect, which refers to how mammary epithelial cells (MECs) interact with their surroundings, has been examined only in recent years (Streuli, 2003). Current genetic analysis has demonstrated that the extracellular matrix (ECM) has a major role in dictating how MECs behave in vivo (Faraldo, Deugnier, Lukashev, Thiery, & Glukhova, 1998; Li et al., 2005; Naylor et al., 2005; Guo et al., 2006).

In the juvenile mammal, the mammary gland consists of primitive ducts, with limited side branching. Upon stimulus by ovarian hormones during puberty, these ducts elongate and branch further into the fat pad surrounding the gland. With pregnancy, the ends of the branches in the ductal system become mature acinar structures, also known as terminal ductal-lobular units (TDLUs) or alveoli (Oakes, Hilton, & Ormandy, 2006). Stromal and myoepithelial cells encompass each duct and alveolus, and provide essential cues for MEC survival, proliferation and terminal differentiation leading to milk secretion during lactation. The fully differentiated alveoli are made of a single layer of luminal MECs encompassing a single hollow lumen, and surrounded by both myoepithelial cells and basement membrane (BM), which is a specialised type of ECM. The myoepithelial cells, and the stroma beyond them, regulate mammary development through the localised secretion of a variety of growth factors (Sternlicht, 2006). Myoepithelial cells have further functions of spatially restricting epithelial cells to form ducts during puberty, mechanically expelling the secreted milk from alveoli, and acting as tumour suppressors (Lakhani & Bissell, 2005).

The BM is established by stromal–epithelial interactions leading to secretion of specific ECM components, and assembly of the 100 nm thick matrix at the basal surface of the epithelium (Kedinger, Lefebvre, Duluc, Freund, & Simon-Assmann, 1998; Streuli, 2006). MECs adhere via several types of ECM receptors, one class of which is composed of hetero-dimeric α- and β-chain integrins (Taddei et al., 2003; Naylor & Streuli, 2006). At least 24 integrin dimers are known, showing a great diversity of ligands (Giancotti & Tarone, 2003; Taddei et al., 2003; Jin & Varner, 2004). An emerging concept is that one of the functions for integrins is to monitor the ECM environment, thereby equipping cells with context-dependent checkpoints for signaling. Thus, integrins only permit growth factor/cytokine responses in cells that are in an appropriate location, providing an important device to maintain normal tissue homeostasis (ffrench-Constant & Colognato, 2004; Guo & Giancotti, 2004; Streuli, 2006).

This review will concentrate on the known effects of ECM on MEC behaviour, particularly focussing on the BM, and the specific integrin signalling events underlying them. We will discuss how integrin-mediated adhesion serves as a checkpoint for different aspects of MEC function through integration with other signals received during mammary gland development.

## 2 Adhesion signalling in the mammary gland

The ECM consists of multiple components, depending on the stroma and epithelium within the specific tissue. The mammary BM contains collagen IV (Col IV) and Laminin I (LM-1, renamed recently as LM-α1β1γ1 (Aumailley et al., 2005)), as well as LM-5, 10 and 11 (LM-α3Aβ3γ2, -α5β1γ1 and -α5β2γ1, respectively). These are cross-linked by nidogen-1 and -2 (also known as entactins) to form a gel-like structure to which MECs adhere (Prince et al., 2002). The mammary stoma secretes a separate set of ECM components such as fibronectin (FN) and tenascins (TNs) (Sakakura, Ishihara, & Yatani, 1991; Schedin, Mitrenga, McDaniel, & Kaeck, 2004; Scherberich et al., 2005).

While some of the studies to dissect ECM function have used purified ECM components, others rely on BM isolated from the Engelbreth–Holm–Swarm tumour (known commercially as Matrigel), which is thought to mimic effectively the mammary BM (Kleinman et al., 1986). There are wide variety of adhesion receptors for BM proteins in the mammary gland, including non-integrin receptors such as dystroglycan, syndecan, galactosyl transferase (Streuli, 2003). However, their specific roles are still not fully clear. Integrin dimers containing the β1 subunit are required for proper mammary gland development (Klinowska et al., 1999; Li et al., 2005; Naylor et al., 2005). Nonetheless, the α-subunits involved in different aspects of mammary function have not yet been defined (Schatzmann, Marlow, & Streuli, 2003; Taddei et al., 2003). Although differentiation signals in vivo require spatial cues from a LM-β1 integrin axis, it is not yet clear which αβ-integrins deliver the key intracellular responses. When genes were deleted independently, two important binding partners of integrin β1 and β4, the α3 and α6 integrin chains, were dispensable in mammary gland development (Klinowska et al., 2001). This indicates some degree of redundancy in MEC integrin function.

Integrins bind ECM proteins when in an extended active conformation, and simultaneously promote the formation of multi-protein adhesion complexes at the plasma membrane (Geiger, Bershadsky, Pankov, & Yamada, 2001; Humphries et al., 2003). Adhesion complexes are both focal centres for assembling the cytoskeleton and signalling platforms for controlling cell behaviour (DeMali, Wennerberg, & Burridge, 2003; Miranti & Brugge, 2002). They function through recruitment of a variety of structural proteins (e.g., talin, vinculin), adaptor proteins (e.g., paxillin, p130Cas, parvin), and enzymes (e.g., integrin-linked kinase (ILK), Syk, focal adhesion kinase (FAK), Src, small GTPases) (Zamir & Geiger, 2001; Cukierman, Pankov, & Yamada, 2002; Brakebusch & Fassler, 2003). The proximal adhesion complex components are well defined in cell types such as fibroblasts and endothelial cells (Gaus, Le Lay, Balasubramanian, & Schwartz, 2006; Pirone et al., 2006), as well epithelial cells in two-dimensional (2D) cultures (Liu et al., 2006). Focal adhesion-like structures formed by MECs on BM (our unpublished data) or in vivo may resemble those in their usage of components such as ILK, Syk, and FAK. Nonetheless, the signal generated by integrins through the focal adhesion depends on the physical ECM properties (Wozniak, Desai, Solski, Der, & Keely, 2003) and may reflect changes in the composition of the signalling complex itself.

ILK binds to β-chains of various integrin dimers and its activation leads to many important events downstream of integrin signalling (Hannigan, Troussard, & Dedhar, 2005; Legate, Montanez, Kudlacek, & Fassler, 2006; Schatzmann et al., 2003). ILK is especially important in inducing cytoskeletal reorganization, a critical event in cell polarization and morphogenesis. Moreover ILK appears to have a key role in the differentiation response of mammary epithelium (R Marlow and CH Streuli, unpublished data).

Another kinase interacting directly with the integrin β-chains is Syk (Dejmek et al., 2005). This kinase participates in activation of hematopoietic cells, integrin signalling and MEC transformation (de Virgilio, Kiosses, & Shattil, 2004; Dejmek et al., 2005; Katz et al., 2005). Similarly to integrin signalling in hematopoietic cells, Syk may induce actin polymerisation via the Vav guanine exchange factor family (Miranti, Leng, Maschberger, Brugge, & Shattil, 1998). It may also be involved in cross-talk with EGFR in MECs (Ruschel & Ullrich, 2004).

The interaction of cells with ECM leads to the formation of focal adhesions, an event that involves FAK. This enzyme is linked to activated integrins through the proteins talin and paxillin (Schatzmann et al., 2003). In MECs, the complex that FAK forms downstream of integrin activation may be separate from that which is formed by ILK or Syk (Miranti et al., 1998). Activation of FAK is intimately related to activation of Src tyrosine kinases. Src kinases are activated by growth factor receptors and may act as a link between them and integrin signalling (Brunton, MacPherson, & Frame, 2004). Moreover, Src is involved with MEC development and proliferation through its effects on estrogen receptor-α (ERα) and the adaptor protein p130Cas (Cabodi et al., 2006; Kim, Laing, & Muller, 2005). In fibroblasts, endothelia and other cell types, FAK activation leads to proliferative signals through the MAPK cascade. An adaptor protein linking FAK to this cascade, Shc, binds only to specific integrin dimers, such as α1β1, α5β1, αvβ3 and α6β4 (Mainiero et al., 1995; Wary, Mainiero, Isakoff, Marcantonio, & Giancotti, 1996).

A pressing question is to decipher the molecular details of how integrins control fate decisions of mammary epithelium, and elucidating the role of specific adhesion complex components will be implicit in this analysis.

## 3 Survival and apoptosis

Perhaps the most general role ascribed to integrin-mediated adhesion is cell survival. In virtually all adherent cells, including MECs, cell detachment from the ECM leads to a type of programmed cell death (or apoptosis) called anoikis (Frisch & Francis, 1994; Gilmore, 2005).

The mechanism for anoikis in MEC has been explained in part, and involves a direct signalling pathway linking integrins to the intrinsic control centre for apoptosis, the mitochondria. The initiating events are poorly understood, but within 15 min of disengaging integrins and the ensuing cell detachment, the pro-apoptotic proteins Bax and Bid translocate to the mitochondrial membrane (Gilmore, Metcalfe, Romer, & Streuli, 2000; Valentijn & Gilmore, 2004; Valentijn, Metcalfe, Kott, Streuli, & Gilmore, 2003; Wang, Valentijn, Gilmore, & Streuli, 2003). As a result, the mitochondrial membrane potential depolarises and cytochrome *c* is released to the cytoplasm. Cytochrome *c*, together with Apaf-1, activates the cysteine protease family of caspases, which cleaves a wide range of protein targets ultimately resulting in cell death (Jin & El-Deiry, 2005).

**Fig. 1:**
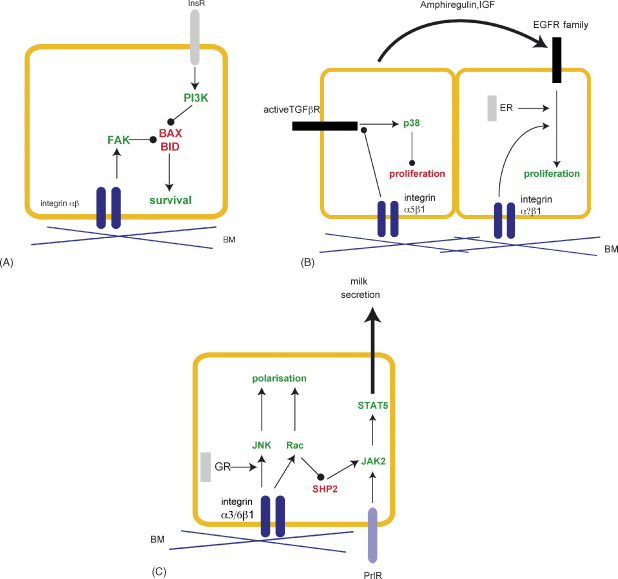
(A) MECs require a basement membrane (BM) checkpoint for survival. Adhesion of MECs through integrins to the BM prevents cells from undergoing anoikis. A pro-survival signal from activated integrin dimers mediated by FAK blocks Bax and BID translocation to the mitochondria. The integrin signal integrates with one from the insulin receptor (InsR) to promote long-term survival. These events prevent cell death, as long as the cell adheres to the BM. (B) The proliferation checkpoint comprises signals from growth factors, hormones and integrins. In the duct, some MECs (left) express integrin α5β1 and this enables a p38-mediated signal from activated TGFβ to inhibit their proliferation. Neighbouring cells (right) express different β1-integrin dimers. As a result, they are receptive to integrated proliferative signals from the EGFR family and the estrogen receptor, while TGFβ signalling is inactive. (C) MEC-BM interaction provides a checkpoint for terminal differentiation. MECs within alveoli require adhesion signals for both polarisation and milk secretion into the lumen. Rac GTPase is central for both events. Together with JNK, it integrates signals from both glucocorticoids and integrins α3β1 and α6β1 to maintain cell polarisation. In another branch of signalling downstream of Rac, the phosphatase SHP-2 may be blocked to enable prolactin-mediated STAT5 phosphorylation and production of milk proteins.

Studies in mammary cell lines have indicated that FAK over-expression can prevent anoikis (Gilmore et al., 2000). Conversely, MEC genetically lacking β1 integrin show increased apoptosis in tissue culture, concomitant with reduced FAK phosphorylation (Li et al., 2005; Naylor et al., 2005). FAK is linked to Rac activation downstream of β1 integrin in fibroblasts (Hirsch et al., 2002) and may function similarly in MECs as well. Moreover, FAK may regulate Bax translocation to mitochondria through a pathway involving PI3K, protein kinase B (PKB/Akt), and the serine–threonine kinase, Pak1 (P. Wang, Q. Pu, C.H. Streuli, unpublished data) (Fig. 1A).

The ECM *per se* is not sufficient for sustained MEC survival, but requires particular matrix ligands. The BM protein LM-1 suppresses anoikis much more efficiently than stromal ECM such as Col I (Pullan et al., 1996), though it is not yet clear whether ECM can trigger survival responses, when presented as individual proteins or if assembly into higher-order matrices is required. Although the survival stimulus partly results from interaction between LM-1 and its receptor, the α6β4 integrin, additional signals from insulin or insulin-like growth factor (IGF) are also required (Fig. 1A) (Farrelly, Lee, Oliver, Dive, & Streuli, 1999). The signalling effects of insulin/IGF, leading to phosphorylation of the adaptor, insulin receptor substrate 1 (IRS1), and activation of the PI3K pathway, are generally much more substantial and persistent when cells are cultured on purified BM than on Col I (Lee & Streuli, 1999). This indicates that ECM adhesion receptors are integrated intracellularly with those for the soluble survival factors (such as insulin and IGF). The integration providing this matrix-specific checkpoint for survival is still in need of further clarification at the molecular level, but may involve combinatorial downstream signals, or physical association of the receptors either directly or within lipid rafts, as occurs in other cell types (Decker & ffrench-Constant, 2004). The ECM-integrin survival axis likely works together with signals from cadherin-mediated cell–cell interactions in the mammary gland (Boussadia, Kutsch, Hierholzer, Delmas, & Kemler, 2002), though this hypothesis received little current attention.

Accumulation of milk in alveoli at the end of lactation leads to a tissue-remodelling process known as involution (Green & Streuli, 2004; Hennighausen & Robinson, 2005; Watson, 2006). Although anoikis contributes to involution, it is unclear whether anoikis in MECs is cell autonomous. In endothelial cells, upregulation of cell death receptors such as Fas ligand and/or downregulation of cell receptor signalling suppressors such as c-FLIP accounts indirectly to anoikis (Aoudjit & Vuori, 2001). This extrinsic apoptotic pathway requires a subset of caspases that are largely independent of mitochondrial membrane potential depolarization (Jin & El-Deiry, 2005). FasL-activated caspases do not play a role in MEC anoikis in vitro (Wang et al., 2003). Nonetheless, apoptosis during involution may include both anoikis and cell death receptors (Clarkson et al., 2006).

The contribution of other cell intrinsic pro-apoptotic proteins to anoikis of MECs is still largely unknown. In human MECs, p53 expression was sufficient to sensitise anoikis in response to inhibition of α3β1-ECM contact (Seewaldt et al., 2001). Evidence in vivo indicates that during involution β1integrin is inactivated (Prince et al., 2002) or that there is down-regulation of both β1 integrin and FAK expression (McMahon, Farr, Singh, Wheeler, & Davis, 2004). Hence, loss of integrin signalling (*i*.*e*., anoikis) may contribute directly to involution. A complementary view suggests that involution occurs as a result of changes in the composition of the mammary BM ((McDaniel et al., 2006) and see below). In this case, changes in specific integrin dimer cross-linking may be sufficient to induce anoikis. For example, such change may involve a switch from β1-containing integrins to alternate dimers, as a result of β1 de-activation (Beauvais & Rapraeger, 2003).

In summary, adhesion to the ECM is an absolute requirement for cell survival. However, since survival is ECM-specific (*i*.*e*., it depends on a BM), we view cell–BM interactions as a positive checkpoint to suppress apoptosis, ensuring that mammary epithelial cells are normally only maintained within ducts and alveoli. This is one of the mechanisms guaranteeing that development occurs normally, leading to the formation of intricate epithelial structures that underlie the function of the tissue. A corollary is that if this apoptosis checkpoint becomes activated autonomously, MEC may acquire the potential to survive within inappropriate ECM locations, such as within stroma or lymph nodes; this is a prerequisite for the more subversive aspects of breast cancer, when tumours become malignant.

## 4 Proliferative hormonal responses

The development of the mammary gland is tightly regulated by a variety of hormones, both metabolic and reproductive (Neville et al., 2002). During pregnancy, estrogen and progesterone are critical for MEC proliferation. Estrogen contributes to ductal elongation and stimulation of prolactin release from the pituitary gland (Brisken, 2002). The formation of alveolar structures and side branching is largely dependent on progesterone and prolactin (Oakes et al., 2006). The combined effects of prolactin and ECM in terminal differentiation are discussed in a separate section below.

An important study has compared the ability of MECs from virgin and pregnant mice to respond to estrogen and/or progestin, when adhered to Col IV. Both hormones were capable of stimulating proliferation in the virgin MECs, but not cells derived from pregnant mice (Xie & Haslam, 1997). Neither cell type proliferates in response to BM adhesion or hormonal stimulation alone (Xie & Haslam, 1997; Haslam & Woodward, 2003). Taken together, this evidence suggests that the ECM assists the MECs to respond in accordance with their developmental stage. Contradicting studies exist regarding the regulation of progesterone or estrogen receptor expression by adhesion to BM. One study found them unchanged even in combination of BM adhesion with growth factors, with or without estrogen (Woodward, Xie, Fendrick, & Haslam, 2000). Another group reported that ERα expression is induced by adhesion to purified BM or its major components, LM-1 and Col IV (Novaro, Roskelley, & Bissell, 2003). Furthermore, antibodies to integrin chains α2 and β1 blocked this regulatory effect. Since both studies have used primary MECs, such discrepancies may result from differing culture conditions and await resolution.

MEC proliferation requires integrin-mediated ECM adhesion because genetic deletion of β1 integrin leads to cell cycle arrest and defective mammary gland development in vivo (Li et al., 2005). Crucially, neither adhesion nor hormones alone are sufficient for MEC proliferation, but their integrated signals are required. Therefore, in this checkpoint, cells proliferate in response to hormonal stimulation only if the adhesion context is permissive.

## 5 Growth factor signalling

A variety of growth factors contribute to the development of the mammary gland, mostly secreted by the surrounding stroma and myoepithelial cells (Sternlicht, 2006). The receptors for most growth factors are of the receptor tyrosine kinase class, which is known to have significant cross-talk with integrin signalling (Brunton et al., 2004; Guo & Giancotti, 2004). Most available evidence for such cross-talk concentrates on four growth factor families: EGF, IGF, TGFβ and HGF.

The receptors for EGF ligands such as amphiregulin (EGFR) and neuregulin (ErbB4) are required for proper alveolar development (Yang et al., 1995; Sternlicht, 2006). Using a normal MEC cell line cultured on purified BM, alveolar development was reproduced using ErbB2 stimulation and shown to be dependent on the MEK/ERK pathway (Niemann et al., 1998). Primary MECs from both virgin and pregnant mice proliferate in response to EGF on any ECM component examined (Xie & Haslam, 1997). More differential effects of ECM components on EGFR signaling are not entirely clear. Col IV, LM and FN (but not Col I) adhesion reduce EGF binding to its receptors, EGFR and ErbB2 (Woodward et al., 2000). Both EGFR protein expression and phosphorylation are higher when MECs adhere to Col I than to purified BM (Lee & Streuli, 1999). In contrast, ErbB2 phosphorylation is more extensive on BM than on Col I. As a result, ERK phosphorylation downstream of EGFR stimulation is weaker upon BM adhesion. Interestingly, blocking integrin β1 in malignant MECs cultured on purified BM reverses EGFR phosphorylation and subsequent MAPK activation (Wang et al., 1998). This differential in ECM responsiveness to EGF signalling is reflected by radically different growth characteristics of mammary cells cultured in 2D environments on stromal ECM with growth in three-dimensional (3D) matrices on purified BM (Muthuswamy, Li, Lelievre, Bissell, & Brugge, 2001).

IGF-I is locally induced within the mammary stroma by systemic growth hormone and is capable of mimicking the effects of insulin in supporting, together with the ECM, survival of MECs (Farrelly et al., 1999; Kleinberg, Feldman, & Ruan, 2000). Adhesion to several ECM components (Col I, Col IV, LM and FN) enhances the combined proliferative effect of EGF and IGF-I. EGF stimulates expression of inhibitory IGF-binding proteins in MECs and this effect is suppressed by adhesion to ECM (Woodward et al., 2000).

TGFβ has a well-documented role in inhibition of MEC proliferation and modulating epithelial plasticity (Bhowmick, Zent, Ghiassi, McDonnell, & Moses, 2001; Ewan et al., 2005). Ectopic expression of α5-integrin leads to an increase in both type II TGF receptor expression and autocrine TGFβ secretion in transformed mammary cells, suggesting a link between integrin and TGFβ (Wang et al., 1999). In other cell lines, normal and transformed, a SMAD-independent TGFβ activation of p38 MAPK depends on integrin β1 (Bhowmick et al., 2001). Nonetheless, MECs adhering to purified BM do not undergo apoptosis in response to TGFβ1 (Pullan et al., 1996). The emerging evidence of interplay between estrogen-induced proliferation and its restraint by autocrine TGFβ (Ewan et al., 2005) leads to interesting questions regarding the role of the ECM in this cell fate checkpoint. It is possible that ECM adhesion enables TGFβ to inhibit hormone-driven MEC proliferation, but not to induce apoptosis in vivo (Fig. 1B).

HGF and its receptor c-Met are involved in branching of mammary ducts in a PI3K-dependent manner (Niemann et al., 1998). HGF induces branching of MECs, in vitro, on both Col I and Fibrin gels (Alford, Baeckstrom, Geyp, Pitha, & Taylor-Papadimitriou, 1998). MECs cultured on Col I and treated with a combination of HGF and progestin show behaviour remarkably similar to that observed in mammary alveoli (Haslam & Woodward, 2003). Conversely, HGF antibodies are sufficient to reverse mammary development induced by estrogen and progestin treatment in vivo. Induction of branching by HGF can be reversed in vitro by inhibition of the proper integrin α chain (α2β1 for Col I and α3β1 for Fibrin). De-activation of β1 integrin is also sufficient to block HGF-induced branching on Col I (Klinowska et al., 1999). Evidence in vivo suggests a unique role of α2β1 integrin in the branching process (Chen, Diacovo, Grenache, Santoro, & Zutter, 2002), although the integration of signals from HGF and/or hormones was not characterized at this level.

Growth factor signalling determines whether MEC proliferate, undergo apoptosis, or form branched structures. However, only cell interactions with the appropriate ECM context, mediated by integrins, allow the proper developmental decision. Thus, although growth factors have a wide variety of effects on cells, ECM adhesion permits a much more specific set of outcomes than might have been predicted from studies using 2D culture on plastic alone.

These collective studies therefore indicate that adhesion provides a key checkpoint to determine the proliferative fate of MEC. Subversion of this checkpoint has disastrous consequences, sometimes leading to cancer. The expression of an integrin dimer, the α6β4 laminin receptor, is altered in breast cancer, contributing to poor patient prognosis (Tagliabue et al., 1998). Integrin α6β4 co-localises and associates with ErbB2 in breast cancer cells (but probably not in normal cells), resulting in a gain of function that enhances proliferation and migration (Falcioni et al., 1997). Recent studies have indicated that suppression of β4-integrin in the context of breast cancer, through shRNA approaches or deletion of its signalling domain, has a profound effect on tumorigenesis by reducing ErbB2 signalling thereby preventing proliferation and survival (Lipscomb et al., 2005; Bon et al., 2006; Guo et al., 2006; Yoon, Shin, & Lipscomb, 2006). Thus, understanding how adhesion checkpoints normally work and how they can become altered in cancer has significant implications for future therapeutic avenues.

## 6 Acinar morphology

Acinar structures in vivo (or alveoli) are made of a dual layer of cells: an internal layer of luminal epithelial cells encompassed in a mesh-like layer of myoepithelial cells (Adriance, Inman, Petersen, & Bissell, 2005). Both primary MECs and mammary cell lines form acinar structures in three-dimensional culture on purified BM. The cells polarise and form a central hollow lumen into which milk will ultimately be secreted. Acinar structures of primary MECs form mostly as a result of cell aggregation and subsequent cell polarisation (Barcellos-Hoff, Aggeler, Ram, & Bissell, 1989; Aggeler et al., 1991). In contrast, mammary cell lines develop acinar structures on BM from a proliferating single cell (Debnath & Brugge, 2005). In vivo, alveoli form as a result of proliferation, aggregation, other changes in cell fate (such as reduction in cell death), or a combination of these factors.

The cellular events that are particular to MECs forming alveoli are only partially understood. In other epithelial systems, cellular polarity and lumen formation on purified BM are closely linked (Straight et al., 2004). Polarity is highly dependent on actin polymerisation down-stream of integrins, which is normally mediated by small GTPases. A candidate effector in the mammary epithelium is ILK, which has a critical role in cell spreading (Filipenko, Attwell, Roskelley, & Dedhar, 2005). Actin polymerisation is achieved by ILK activation of β-parvin and its subsequent interaction with α-PIX, a guanine nucleotide exchange factor for both Rac and Cdc42. The contribution of these small GTPases to acinar morphology (and milk synthesis) has recently been described (Akhtar & Streuli, 2006). Rho itself was proposed to be a sensor of ECM rigidity and therefore to determine the morphological fate of MECs (Paszek & Weaver, 2004; Wozniak et al., 2003). On flexible ECM, Rho activity is low and tubules resembling ducts are formed. On a rigid ECM, Rho activity is high and MECs proliferate. Although such studies have been undertaken in mammary cell lines, this may be an attractive explanation for the way the ECM dictates whether ductal or alveolar structures will be formed, if ECM rigidity indeed changes during mammary development in vivo.

Systemic glucocorticoids also contribute to acinar architecture (Fig. 1C). Without hydrocortisone, several stages in acinar morphology do not occur, including cell polarisation and tight junction formation. A single signalling pathway was implicated downstream of glucocorticoids, the MEKK4/JNK pathway, induced by BRCA1/GADD4β (Murtagh et al., 2004). In these studies, inhibition of the JNK pathway was sufficient to disrupt proper integrin β4 expression and lumen formation, two events that are likely to be related to one another.

Polarised acinar structures are key components of the mammary gland, allowing it to deliver milk to infants. We suggest that integrin signalling through small GTPases provide an essential polarisation check point. Without these integrators of adhesion function, actin polymerisaton is absent and acinar structures do not form properly. The JNK pathway is also required for this process, thus only coordinated signals from the ECM and glucocorticoids result in lumen formation.

## 7 Terminal differentiation

Prolactin drives both alveolar proliferation and differentiation during pregnancy, and the subsequent secretion of milk into the alveolar lumen (Oakes et al., 2006). However, an appropriate ECM is also required for proper mammary differentiation and milk secretion in response to prolactin stimulation (Streuli, Bailey, & Bissell, 1991) (Fig. 1C). Downstream effectors of prolactin, Jak2 and Stat5, are activated only when MECs adhere to purified BM but not to Col I (Edwards et al., 1998; Lee & Streuli, 1999). A protein-tyrosine phosphatase is triggered upon adhesion to Col I, but not purified BM, inhibiting prolactin signalling, probably by direct interaction with Jak2 (Edwards et al., 1998). This phosphatase may be SHP-2 (Akhtar & Streuli, 2006).

Mice genetically lacking functional β1-integrin can develop alveoli, although with disrupted morphology and lack of Stat5 nuclear translocation in response to prolactin stimulation (Li et al., 2005; Naylor et al., 2005). Two separate studies attributed the structural abnormalities to alterations in both proliferation and apoptosis in the modified mammary gland (Faraldo et al., 1998; Li et al., 2005). How the loss of integrin β1 directly contributes to defective milk secretion is not entirely understood. It is probably through a control on prolactin-stimulated signalling, via the integrator of adhesion signalling, Rac1. Expression of dominant negative Rac1 leads to disruption of prolactin signalling in MECs adhering to BM. Moreover, β1^−/−^ MECs had reduced Rac activity, and constitutively active Rac1, was sufficient to rescue the β1^−/−^ MEC phenotype and differentiation (Akhtar & Streuli, 2006). Interestingly, Cdc42 activity is required for a distinct morphological process, which is regulated by hormones and not by BM adhesion. This process is not yet defined but may relate to the establishment of cell polarity (Etienne-Manneville, Manneville, Nicholls, Ferenczi, & Hall, 2005; Filipenko et al., 2005).

Milk secretion and cell polarisation are closely linked in the mammary gland. The ECM checkpoint enables MECs to respond to prolactin signaling only when there is a signal through BM-binding β1-integrin dimers, thereby coordinating the temporal aspects of differentiation with the spatial requirement for an organised alveolar architecture. This link is established through integrin-containing adhesion complexes and Rac1 to allow prolactin-driven Stat5 nuclear translocation and the resulting milk synthesis.

## 8 Modulation of ECM composition and its relation to integrin signalling

The ability of integrin signalling to dictate the terminal differentiation of MECs has been known for many years (Medina, Li, Oborn, & Bissell, 1987; Streuli et al., 1991). Perhaps surprisingly, it only emerged later that the composition of the BM itself may change during mammary gland development and assist in this process (Talhouk, Bissell, & Werb, 1992). Although the levels of critical BM components such as LM and nidogens remain constant during pregnancy and lactation, the ECM is modified upon induction of involution, and ligand-bound β1-integrin becomes deactivated (Prince et al., 2002). This partly occurs through the action of matrix-degrading metalloproteinases (MMPs) and serine proteases (Fata, Werb, & Bissell, 2004; Green & Lund, 2005). In ECM isolated from mammary glands at various developmental stages, there is evidence for degradation of both LM and FN during involution (Schedin et al., 2004). These changes are attributed to increased MMP activity and may affect β1-integrin signalling. Interestingly, ECM isolated from involuting mammary glands (or FN fragments) were sufficient to induce apoptosis in a normal MEC line, suggesting that altered ECM composition may actively promote apoptosis. Down-regulation of TN during involution (Schedin et al., 2004) suggests that at least stromal ECM expression is controlled to avoid the promotion of carcinogenesis (Scherberich et al., 2005). Specific fragments resulting from MMP degradation of LM-5 have particularly interesting properties. During involution, the DIII domain of LM-5 is generated from 3.5 EGF-like repeats in the LM γ2-chain. This domain has the ability to bind to EGFR and stimulate the MAPK cascade as well as cell motility and further MMP activity (Schenk et al., 2003). As stromal cells should also be responsive to this effect (Sternlicht et al., 2005), DIII and similar LM fragments may play an unexpected role in directing mammary gland remodelling during involution.

Lastly, integrin expression itself might be regulated by BM adhesion. Early evidence suggested an overall down-regulation of integrin expression, including the β1 and β4 integrins, by purified BM (Delcommenne & Streuli, 1995). A more specific observation suggests that nidogen-1 augments the ability of LM-1 to induce mammary epithelial differentiation (Pujuguet et al., 2000).

Taken together, these findings suggest that BM modulation may contribute by itself to the type of integrin signalling in each developmental stage of the mammary gland, thus influencing the checkpoints for MEC fate.

## 9 Perspective: integration of integrin signalling

Integrin signalling is a critical component of the temporal–spatial development of the mammary gland. While studies discussed here demonstrate that this tissue relies on integrins for its function, many questions are left unanswered. (1) Which of the precise integrin dimers mediate each of the functions discussed above: survival, proliferation, morphogenesis and differentiation? (2) How do the integrin-mediated signals result in proper terminal differentiation and milk secretion, and in the process of involution that follows it? (3) Which precise components of the integrin-containing adhesion complex are involved with the different checkpoints? (4) What is the role of integrins on maintaining the stem cell niche? (5) How does local ECM remodelling by membrane-bound proteases affect integrin signalling and thereby cell fate? (6) What is the influence of integrins on other cell types within the mammary gland, including stromal cells and myoepithelial cells? (7) To what extent to the less-well studied ECM ligands and receptors influence integrin signalling?

To achieve this level of understanding more research into the molecular mechanisms of signal integration is required. For example, anecdotal evidence shows that FAK and ILK are probably involved as scaffold proteins in integrin signaling as much as they are as kinases (Schatzmann et al., 2003) and may serve as a bridge between integrins and different receptors such as those for EGF (Wang et al., 1998). Nonetheless, a long list of other candidate adaptors includes paxillin and Nck-2, among others (Zamir & Geiger, 2001).

The issue of signalling events underlying acinar morphogesis is particularly intriguing. Evidence regarding the involvement of Rho GTPases begins to shed light on the processes that may be involved (Akhtar & Streuli, 2006). Nonetheless, we are far from a full understanding of how ECM signalling ultimately links to other critical cellular elements such as gap and tight junction communication (El-Sabban et al., 2003; Li et al., 2005), or to desmosomal adhesion (Runswick, O’Hare, Jones, Streuli, & Garrod, 2001).

Although genetic systems, such as *Cre-Lox* gene deletion and the use of shRNA, have now become tractable for dissecting these questions both in culture and in vivo, better models will be of key importance. Particular challenges will be the development of 3D primary cell culture models for studying in vivo-like branching morphogenesis, the stem cell niche, alveolar formation from ducts, and luminal–myoepithelial–stromal cell interactions, as well as animal models for live cell imaging and genetic targeting of both stem cells and stromal cells.

In this review we have attempted to demonstrate the interdependency of tissue architecture and cell fate decisions in mammary gland development and function. Continuing efforts in the area of signalling integration will undoubtedly be of outmost importance.
